# Supplementary figures and images for: Artificial Extracellular Vesicles Generated from T Cells Using Different Induction Techniques
Source: Biomedicines. 2024 Apr 20;12(4):919. doi: 10.3390/biomedicines12040919 (PMC11048032; doi:10.3390/biomedicines12040919)

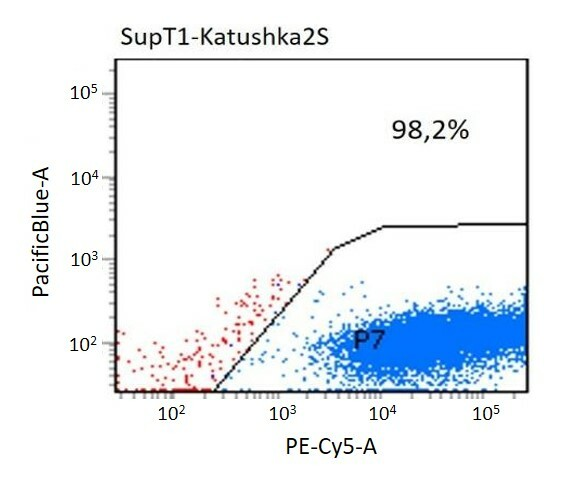

Supplement: Supplementary file 1 [file biomedicines-12-00919-s001.zip › FigureS2.tiff]

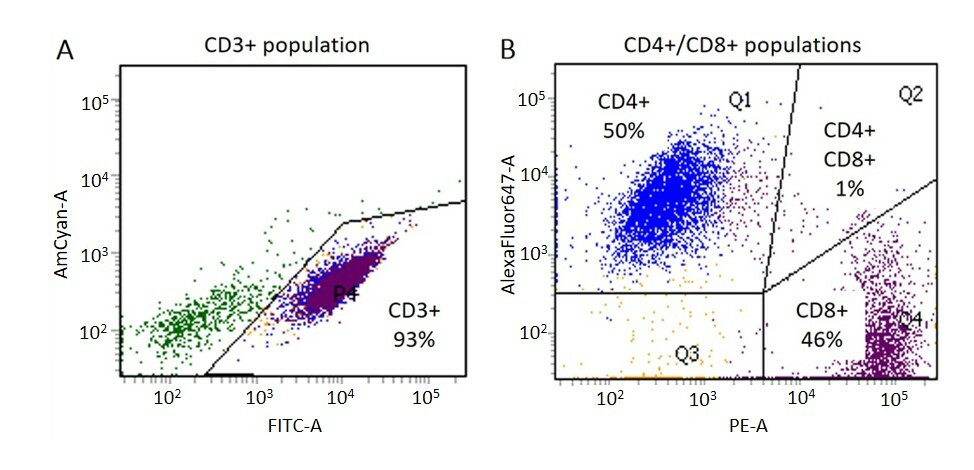

Supplement: Supplementary file 1 [file biomedicines-12-00919-s001.zip › FigureS1.tiff]
